# Do smart services promote sustainable green transformation? Evidence from Chinese listed manufacturing enterprises

**DOI:** 10.1371/journal.pone.0284452

**Published:** 2023-04-27

**Authors:** Yan Chen, Bin Xu, Yuqi Hou

**Affiliations:** School of Economics and Management, Beijing University of Posts and Telecommunications, Beijing, China; Hainan University, CHINA

## Abstract

Smart services are expected to solve the dilemma of development and emission reduction, but there is still no conclusive evidence on whether and how they work. This article aims to explore the relationship between smart services and sustainable green transformation and the effect mechanism. To achieve this goal, a text mining analysis is performed to assess 970 Chinese listed manufacturing enterprises’ smart services development; a regression analysis is then conducted. The results show that smart services have a significant positive impact on the quality and quantity of green innovation, especially for heavily polluting enterprises. The substitution of technology and labor for capital, as well as the upgrading of human resource quality, are effective mechanisms. Smart services can assist as a management strategic tool to balance environmental protection and development, but such an effect fails to work in areas not covered by new infrastructure and is weaker for private enterprises.

## 1. Introduction

China is the largest industrial carbon emissions country in the world. According to the latest data from the Organization for Economic Co-operation and Development (OECD), China’s industrial carbon emissions account for 34.7% of the world’s total, 3.1 times that of the United States and 3.5 times that of the European Union. Massive carbon emissions directly aggravate the greenhouse effect, leading to serious consequences, including sea level rise, extreme climate disasters, desertification, ocean acidification, and the occurrence and spread of infectious diseases. It endangers human survival and the sustainability of the natural environment. China’s goals of achieving a carbon peak by 2030 and carbon neutrality by 2060 are of great significance to human survival and natural sustainability. The time limit for this goal forces the industrial sector to accelerate the green revolution in China. However, during this implementation process, extreme phenomena have occurred, such as campaign-style “carbon reduction”, “one-size-fits-all”, and even “power rationing”. These significantly deviate from high-quality development, which is the essential requirement of the “dual carbon” goals. It is urgent to solve the dilemma of development and emission reduction and find a path that can balance environmental protection and development for the manufacturing industry by focusing on the transformation of the development mode [1–3].

**Fig 1:**
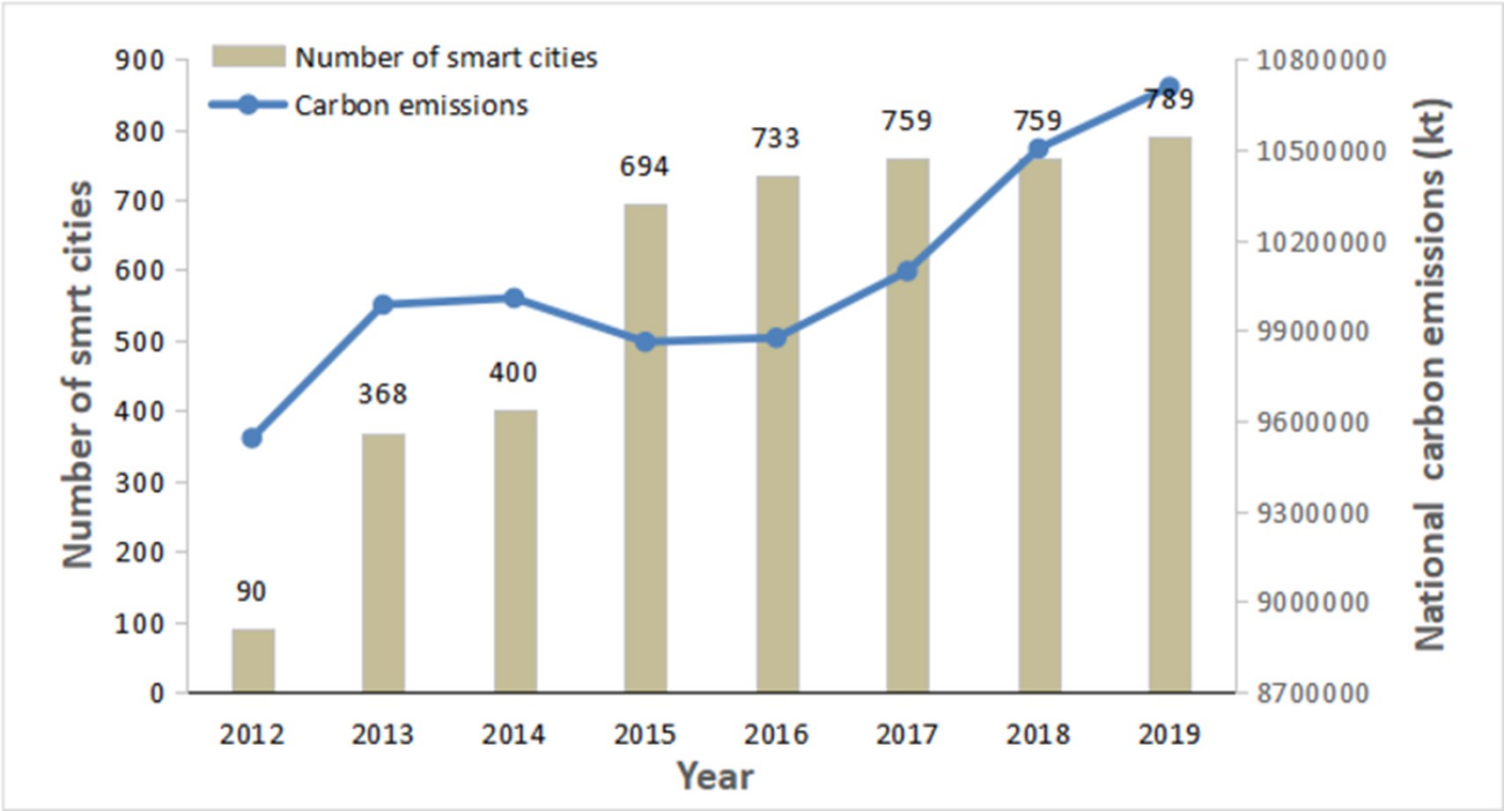
Number of smart cities and national carbon emissions in China. Source: Author´s design based on data from the Chinese government public information; Word Bank, 2022.

Governments around the world are expected to achieve environmentally sustainable economic growth by adopting policies that promote green economic activities. Some have achieved positive results, such as financial market deepening, trade openness and globalization [4–6]. However, some studies suggest that economic activities may have negative effects on sustainable development [7,8]. It can be concluded that economic activities directly affect green development, but their effects must be further examined. Smart services provide a possible research perspective for attaining this goal. Under the background of the “dual carbon” goals, China tries to transform its development mode by developing advanced manufacturing industries to decouple economic growth from energy consumption. Their application scenarios are widely covered in smart cities, smart manufacturing, smart logistics, smart transportation, smart homes, etc. [9]. Statistics show that in China, the number of smart pilot cities has grown to 789, as shown in Fig 1. The annual output value of smart manufacturing has exceeded 250 million yuan and will maintain an annual growth rate of 15%. There are approximately 160 million users of smart connected vehicles, with an industry penetration rate of over 50%. Approximately 270 million smart home devices are sold annually, accounting for 60% of the global market share.

The development of smart services follows market rules and has incentives associated with a business model [10,11]. As an important form of service innovation, enterprises provide contextualized, personalized and dynamic digital service solutions to users through products with sensing, control, optimization and autonomy functions in smart service systems. Smart services are a bundle of physical products and digital services [12,13]. This changes the way services are allocated and provided, with the potential for substitution of capital and labor [14,15]. Additionally, products with smart consciousness collect and process their own situational data and other required data into smart data, which are put into the cycle of co-creation of sustainable development value as new production factors [9,16]. Therefore, detecting whether smart services have a green transformation effect and exploring the mechanism from the perspective of production factor substitution can solve the current external policy failure at the source. It can provide guidance for manufacturing enterprises to achieve coordinated economic and environmental development under the "dual carbon" constraint.

This study seeks to empirically explore the effect of smart services on sustainable green transformation and its effect mechanism. We test our hypotheses using econometric analysis on a sample of 970 Chinese listed manufacturing enterprises. Based on a measurement index of smart services by a text mining method, a multiple linear baseline regression model and mediating effect regression model are established. The analysis reveals that smart services have a significant positive impact on green transformation, especially for heavily polluting enterprises. The substitution of technology and labor for capital, as well as the upgrading of human resource quality, are effective mechanisms. The effect has regional heterogeneity and is not significant in areas not covered by new infrastructure. Compared with state-owned enterprises, the effect of private enterprises is weaker. Moreover, we conduct robustness tests by constructing instrumental variables (IV) and using the two-stage least square (2SLS) method, as well as alternative measures of independent and dependent variables. The results show that the conclusion is robust.

The main contributions of this paper are as follows. First, the overall attention given to research on smart services and sustainable green transformation is on conceptual frameworks, models and theoretical evaluations [1,17,18]. The few existing empirical studies are mainly conducted at the regional level [19,20], and their research conclusions fail to reach consensus. There is a lack of quantitative research at the micro level, which hinders evidence-based understanding of the phenomenon. In this study, the enterprise green transformation effect of smart services is empirically tested as a business model innovation. It provides a path for enterprises to solve the dilemma of development and emission reduction. Second, the existing discussion of production factors is mainly focused on economic issues [14,15,21]. Although some scholars have given attention to the green effect of production factors, they only discuss the direct effect of production factors on energy consumption [22–24] and ignore the important role of substitution between production factors. There is a gap in the research on smart services and green transition with production factor substitution as the intermediary mechanism. In this paper, we apply the theory of production factors to the study of smart services and green transformation and reveal the substitution of production factors. The mechanism of production factor substitution is explored, and the problem of external policy failure can be solved from the source. Third, relevant studies only discussed the overall green effect, ignoring the conditional effect of regional and firm heterogeneity factors, such as new infrastructure. This study detects the effects of heterogeneity, providing guidance for managers and governments to balance environmental protection and development in a more comprehensive way.

The rest of the paper is structured as follows: Section 2 reviews the related literature, Section 3 presents the hypothesis, Section 4 describes the data and methodology, Section 5 presents the main results and discussion, and Section 6 presents the research conclusions.

## 2. Literature review

### 2.1 Smart services

In a broad sense, services provided based on the new generation of smart technologies and smart connected products are collectively referred to as smart services; these comprise big data analysis services, cloud computing services, and Internet of Things services. Early studies used “teleservice”, “electronic service (e-service)”, and “digital service” to describe the phenomenon of smart services. In 2005, Allmendinger and Lombreglia proposed that smart services should go beyond maintenance and upgrading that may be bundled with products and emphasized the necessity of manufacturing to provide smart services through smart products and thus realize value-added for customers and enterprises [11]. Since then, "smart services" have been gradually accepted and widely used in academia [25].

Teleservices are based on information and communication technology. E-services and digital services accomplish their tasks based on internet-based information resources, applications or digital transactions. Smart services are distinct from them. Smart products (smart devices) with smart awareness, such as sensing, connecting and driving, collect environmental data and process it into smart data. Smart services are scenario-related and demand-oriented personalized solutions provided through digital platforms within smart service systems, thus creating added value for suppliers and customers [26–28].

A smart service is a service system with technology mediation, continuity and program interaction [29], which is described as smart city, smart home, smart health care, smart transportation, etc., in different applications [9]. As the boundary object of the service system, smart connected products with monitoring, control, optimization and autonomy integrate the resources and activities of system participants [30]. Services are provided to both service providers and users through interactions. Therefore, the dynamic allocation of resources and value cocreation between service providers and users are realized [31,32]. The insights brought by the exponential data value of smart services completely change the competitive landscape [16,33]. To achieve this, enterprises make efforts to reform their strategies, business models and resource management [34,35].

### 2.2 Smart services and sustainable green transformation

Some scholars have given attention to the positive relationship between smart services and green transformation, mainly discussing conceptual frameworks, models, and theoretical assessments. Smart services can support better and more professional decision-making processes [36] and can further provide sustainable solutions by optimizing processes [37]. Langley pointed out that the business model of smart services could be in line with sustainability strategy [10]. Enterprises voluntarily extend the service life of products through life-cycle management, which is beneficial to the environment in terms of energy use and emission reduction. Smart services promote information transparency and the process cycle based on the collection, transmission, storage and analysis of data and thus realize the efficiency improvement of products in use, maintenance, reuse and remanufacturing and promote the circular economy [17].

Smart cities and services have been closely related to the practical problems of urban development since their inception. Alberto regarded smart cities as a new type of city that integrates green and social development [38]. Guo, Wang, and Dong empirically researched the impact of China’s smart city pilot policy on energy conservation and emission reduction and found that smart city construction significantly reduced per capita carbon emissions [19]. However, there are some studies that clearly note that sustainability is not originally the primary goal of the development of smart services [39], and its frame design lacks environmental awareness [40]. Despite being the world’s first smart city, Songdo New Town in Korea still had no clear sustainable effect after more than a decade of investment [41], and even environment negative externalities were found [42]. Tan and Md took 15 smart cities in the UK as a sample and found that the relationship between the smart degree of a city and carbon emissions is nonlinear [20].

### 2.3 Substitution of production factors

Regarding the change in production factors caused by smart services, existing research focuses on the substitution of technological progress for capital and labor. Graetz and Michaels suggested that artificial intelligence (AI) substituted labor by using cheaper capital [14]. However, some studies have proposed that the implementation of AI and automated production technology directly substitutes labor factors [15,21]. In essence, automated production does not substitute all the labor of the enterprise. For positions in enterprises that are difficult to code for automation, smart service technology helps improve production efficiency [43]. These studies mainly discuss macroeconomic issues such as employment [14,21,44], productivity and economic growth [15,43] through changes in production factors caused by smart services.

The input structure of production factors is the most direct factor affecting energy consumption. The literature on the elasticity of capital and labor substitution focuses on the relationship between factors of production and energy intensity. Berndt and Wood studied the data of the manufacturing industry in the United States and found that there was a clear complementary relationship between energy and capital and a slight substitution relationship between energy and labor [45]. Popp pointed out that the relationship of labor and energy as well as that of capital and energy were all complementary [22]. However, some scholars hold different views on the relationship between them. They proposed that there was a significant substitution relationship between energy and capital as well as between energy and labor, and the substitution effect of labor was more obvious. More capital or labor substituted for energy can effectively reduce carbon emissions [23,24].

Throughout the literature, the relationship between smart services and sustainable green transformation mainly focuses on qualitative research but lacks quantitative research. This impedes the evidence-based understanding of the phenomenon. The few existing empirical studies are mainly at the regional level, and their research conclusions fail to reach consensus. Whether smart services affect the sustainable green transformation of enterprises is still unclear. This hinders evidence-based understanding of the phenomenon. From the perspective of factors of production, the existing discussion is mainly used to explain economic issues such as production function and endogenous economic growth model. Although some scholars have given attention to the green effect of production factors, they only focused on the direct effect of production factors on energy consumption and ignored the important role of the substitution relationship between production factors in energy conservation and emission reduction. There is a gap in the research on smart services and green transition with production factor substitution as the intermediary mechanism. In addition, relevant studies only discussed the overall green effect, ignoring the conditional effect of regional and firm heterogeneity factors, such as new infrastructure.

## 3. Hypothesis development

### 3.1 The effect of smart services on sustainable green transformation

Smart services are considered a new mode of servitization [46]. Servitization effectively reduces the energy consumption intensity of enterprises, but it also has negative externalities [47]. As a new business model, smart services can overcome the negative externality of the traditional servitization mode to the ecological environment. Enabled by new technologies, smart services are paid for use by product tracking. It avoids the negative environmental impact caused by the irresponsible behavior of users under traditional servitization modes, such as product leasing. Since customers pay for services by usage, companies have a stronger incentive to offer more efficient products to reduce the operating costs associated with product usage. More efficient products are green because they consume less energy and resources in the process of being used. More importantly, precise payment based on usage can inhibit consumers’ use frequency of high-efficiency products to a certain extent, which can effectively alleviate or even solve the problem of the “rebound effect” [1].

Furthermore, the positive environmental effects of smart services as a new business model are prominent. First, smart services collect environmental data and process it into smart data through products with smart awareness, such as sensing, connecting and driving. Context-relevant and demand-oriented solutions, within smart service systems, are provided to users through digital platforms [11,26,33]. Therefore, smart services are greener and energy saving. For example, Toyota has implemented an eco-driving service that intelligently optimizes driving route selection and driving behavior, resulting in significant positive environmental effects in terms of fuel savings and reduced emissions [48]. Second, smart services adopt full life cycle management, in which enterprises monitor and evaluate the current situation in real time, predict potential failures and proactively arrange maintenance and upgrade activities. By continuously optimizing products and improving the quality performance and reliability during the life cycle of such products, the service life of these products is extended so that enterprises can minimize waste and produce positive environmental effects [49]. Third, smart services data provide unprecedented insights into the use and operation of products and user characteristics, which effectively promote the subsequent implementation of ecological cycles such as product reuse, remanufacturing and recycling [17]. As the incentive of the business model is in line with the principle of the circular economy [10] and improving environmental performance means enhancing competitiveness under the restrictions of the government and consumers, smart services become the internal driving force of sustainable green transformation for enterprises [50].

Considering the above, we propose the following:

**Hypothesis 1:** Smart services have a positive effect on enterprise sustainable green transformation.

### 3.2 The mediating mechanism of the effect of smart services on sustainable green transformation

The perspective of production factors is one of the important research perspectives on enterprise issues. The manufacturing process is the basis and core of the output efficiency and profit increment of manufacturing enterprises. However, the inner operation mechanism between input and output is relatively recessive, so it becomes the focus of the "black box" of enterprises. By separating production factors such as land, capital and labor and expanding to new production factors including technology, knowledge, entrepreneurial talent and data, the "black box" of enterprises has gradually opened. According to the theory of production factors, excluding direct natural resources, the factors of production of enterprises in the digital economy mainly include labor (L), capital (K), technology (T) and data (D). The corresponding production function is

Q=f(L,K,T,D) (1)

where *Q* is the output in the broad sense and *f* represents the functional relationship between the factors of production and output.

As the input of the production process, factors of production are the source of energy consumption of enterprises. The development of smart services may lead to the substitution of factors of production, thus affecting the sustainable green transformation process of enterprises.

With the development of smart services, the input of capital may be substituted by technology. As an important form of service innovation, smart services are the collaborative combination of physical products and digital services. Enterprises deliver digital services to users through products and provide users with demand-oriented personalized solutions in the smart service system [12,13,26]. This changes the way services are configured and provided [51]. As functionality moves from the mechanical parts of the product to the software, the physical complexity of the product decreases. This shift reduces the onerous production steps required to build and assemble physical components and reduces capital inputs such as machinery, equipment and raw materials. These are substituted by technology inputs, such as embedded software, cloud platforms, gateways, business integration systems, etc. [16]. In addition, technological progress changes the production efficiency of factors and affects the input ratio of the factors of production. A smart service is a technology-enabling service that integrates multiple technical capabilities [52]. As extension technologies for production factors, these new technologies directly substitute the traditional mode of production and thus substitute both labor and capital [53,54]. Since the traditional development mode of smart service enterprises is capital intensive, smart technology is more inclined to substitute for the capital of enterprises.

The capital of enterprises mainly includes machinery, equipment, plants and raw materials, which are material inputs. Their energy consumption is much higher than their technology consumption. Therefore, smart services have a positive effect on energy savings and emission reduction and promote the sustainable green transformation of enterprises through the substitution of technology for capital. The following hypothesis is proposed:

**Hypothesis 2:** Smart services promote the sustainable green transformation of enterprises through the substitution of technology for capital.

For manufacturing enterprises, the implementation of smart services effectively promotes their extension to both ends of the value chain. Compared with processing, manufacturing and assembly, enterprises use more human capital at the high end of the value chain, consume less energy, have low carbon emissions and do little damage to the environment. It can be seen that capital factors are directly substituted by labor factors, which has the effect of saving energy and reducing emissions.

In addition, the capital of smart service enterprises may be indirectly substituted by labor factors. Smart services use data as the core of reform [16]. Data instead of material becomes the key input factor. Data are consumption-free, reusable and renewable. As a factor of production input, data can promote green and sustainable development by substituting material factors of production. However, the data itself cannot become a factor of production and generate value spontaneously. Its value creation mainly depends on people’s ability and wisdom [55]. Human-computer collaboration is the most important link in the process of data value discovery, including data selection, big data algorithms, high-level semantic understanding, management decision participation and decision quality evaluation. Therefore, labor indirectly substitutes for the input of capital through the synergistic effect of data.

In summary, capital is directly or indirectly substituted by labor with lower energy consumption. Therefore, smart services have a positive effect on energy savings and emission reduction and promote the sustainable green transformation of enterprises through the substitution of labor for capital. The following hypothesis is proposed:

**Hypothesis 3**: Smart services promote the sustainable green transformation of enterprises through the substitution of labor for capital.

The substitution of smart service technology for labor is structural. While low-end labor is reduced, high-end labor that is difficult to substitute with machine intelligence is increased [56,57]. The development of human resources enables enterprises to have more high-end talent, stronger independent innovation ability and the ability to absorb foreign technology. These enterprises improve the efficiency and reliability of production processes and product performance, thus reducing the adverse impact on the environment. Moreover, high-end talent with different expertise working together can inspire each other, and the collaborative innovation of green and low-carbon technologies can be promoted through the spillover effect of knowledge. In addition, with the improvement of human resources, operations and management become more efficient, and thus realize the optimal allocation of resources, eliminate redundancy, reduce energy consumption, reduce waste, and promote the transformation of enterprises to green and lean. Thus, we propose the following:

**Hypothesis 4**: Smart services promote the sustainable green transformation of enterprises through the human resource upgrading effect.

## 4. Research method

### 4.1 Samples and data collection

The samples in this study are from China’s manufacturing industry, whose classification code (GB/T4754-2017) is C34-C40, including (1) general equipment manufacturing; (2) special equipment manufacturing; (3) automobile manufacturing; (4) railway, shipbuilding, aerospace and other transportation equipment manufacturing; (5) electrical machinery and equipment manufacturing; (6) computer, communication and other electronic equipment manufacturing; and (7) instrument manufacturing. These industries are selected as research objects because their products and services meet the prerequisite for developing smart service business. Enterprises in these industries have a more obvious tendency to develop smart services and are at the forefront of the reform. International practice shows that the major players in these industries, such as Xerox, Siemens, Tesla motors, Airbus, Philips and Hewlett-Packard, are the first to adopt smart service strategies. Smart services have become a new business model for these enterprises and are a key part of their revenue, some of which even exceeds the revenue from goods sales. According to the special action of developing service-oriented manufacturing proposed by the Ministry of Industry and Information Technology of China, the industries of the sample enterprises are the key development fields of smart services in China.

We collect the annual reports of all C34-C40 manufacturing enterprises listed in the Shanghai and Shenzhen stock markets in 2019. By checking the text manually, interference information is removed to ensure the clarity and accuracy of the sample enterprises implementing smart services. The missing information in the annual report is verified and supplemented according to the official website and official news. Enterprises with ST, *ST, PT, delisted enterprises, and enterprises with missing important data are excluded. Finally, 970 enterprises are obtained as research samples.

Annual reports are downloaded from the cninfo network. Data on enterprises are sourced from the WIND database, CSMAR database and CNRDS database. City-level data are collected from the China Smart City Yearbook.

### 4.2 Smart service measure index construction

The text mining method has been adopted in the study of economics and management to construct indicators based on the frequency of keywords. The higher the frequency of keywords, the more attention enterprises attach to the objects represented by keywords, and the higher the degree of the object represented by the keywords in the enterprise. As an important strategy to enhance the competitiveness of enterprises, relevant information on smart services should be disclosed in the annual reports of enterprises. Based on the annual report, it is feasible and scientific to describe the degree of smart services from the perspective of the smart service keyword frequency using the text mining method.

According to the characteristics of the information disclosed in the annual report, we set up the keyword database based on the definition of smart service, which is described as smart city, smart home, smart health care, smart transportation, etc., in different applications [9,29]. We summarize the practical application of smart services based on a series of studies [9,58–60] as well as important policy documents and research reports in China. The expression of smart service systems in separate business applications is determined through the combination of Python algorithm automatic word segmentation and manual processing. On this basis, the selected keywords are identified and checked through semantic analysis. The final keywords selected are shown in Table 1.

**Table 1:** Keywords of smart services.

smart service systems
smart city
smart home/kitchen; food networking
smart medical care/health care/rehabilitation/pension
smart transportation/travel/driving/parking/station/subway/driving test; internet of vehicles
smart agriculture
smart manufacturing/factory/production line/production/processing/packaging/sorting; the industrial internet
smart logistics/freight/shipping/storage/warehouse/port
smart education/campus/classroom
smart energy/mine/wind farm/gas/water supply/water conservancy; energy internet
smart power/grid/power plant/distribution/micro electricity/power transformation/electricity consumption/lighting; pile networking
smart security/emergency/fire/coastal defense/command and dispatch/monitoring
smart environmental protection/environment/sanitation
smart government affairs/public security/judicial/police affairs/urban management/people’s livelihood
smart business/finance/bank/broadcasting and TV/retail/culture and tourism/catering/business hall; clothing networking
smart audiovisual/display/meeting
smart metering/calculation
smart community/industrial park/buildings
smart application/business/ecology

Note: The actual index includes synonyms (such as wisdom and intelligence) and English abbreviations to avoid omissions caused by expression and format problems.

On this basis, we use Java PDFbox to extract the text content of the annual report, then search, match the keywords, and count the word frequency. Considering the dynamic competitive environment, we take the reciprocal of the total keyword frequency of all enterprises in the same industry as the measure weight. The calculation formula of the smart service degree is as follows:

$${SmartSer}_{i}=k_{i}/\sum_{j=1}^{n}k_{j}$$ (2)

where *SmartSer*_*i*_ represents the smart service degree of enterprise *i*; *k*_*i*_ represents the frequency of smart service keywords in the annual report of enterprise *i*; and *n* represents the number of enterprises in the industry to which enterprise *i* belongs.

### 4.3 Other variables

We use sustainable green transformation as the dependent variable. There are three main measurement methods of sustainable green transformation in existing studies, including green total factor productivity (GTFP), evaluation index systems and green innovation. GTFP covers both economic and environmental benefits. However, due to the availability of actual data, the expected output is estimated by the regional proportion of economic indicators such as output value. The undesired output uses the data of industrial pollutant emissions, so the environmental impact measured by GTFP only covers industrial production, ignoring the environmental impact of other important aspects, such as product use. The index of the evaluation index system is more systematic and comprehensive. However, the analytic hierarchy process (AHP) adopted is based on the subjective judgment of experts. Therefore, there are shortcomings in the objectivity of the index system. Green innovation is the core of sustainable green transformation, which not only has the commercial characteristics of improving competitiveness but also has the environmental characteristics of energy conservation and emission reduction. Compared with the previous two indices, green innovation can compensate for the above deficiencies. Green innovation covers product design, production, consumption and recycling, so the environmental impact is comprehensively measured from the perspective of energy savings, emission reduction and sustainability. The accessibility of innovation patent data ensures accuracy and objectivity.

Therefore, we select green innovation as the proxy variable of sustainable green transformation. Considering the innovation difference of different types of green patents, green innovation quality (*GreInvia*) is measured by the number of green invention patent applications of enterprises, and green innovation quantity (*GrePatent)* is measured by the total number of green patent applications. The data are processed by natural logarithm. In addition, considering the lag of the sustainable green transformation effect of smart services, this paper adopts data with one lag period to conduct model regression in the selection of dependent variables to avoid immediacy bias.

We select variables related to dependent variables as control variables. Enterprise size (*Size*) is taken as the representative variable of enterprise characteristics. Cash holdings (*Cash*), asset-liability ratio (*Debt*) and return on assets (*ROA*) are used as the measurement variables of the financial status of the enterprise. The governance structure of an enterprise is measured by the state-owned component (*State*). The R&D status of an enterprise is measured by R&D intensity (*RI*). Meanwhile, to control the impact of industry heterogeneity, the Herfindahl-Hirschman Index (*HHI*) is used to measure industry competitiveness. The intensity of industrial policy *(IndPolicy*) is measured by the government subsidies received by enterprises to control the impact of industrial policy. The names and definitions of the variables are described in Table 2.

**Table 2:** Variable description.

Variable name	Symbol	Description
**Dependent variable** Green innovation quality Green innovation quantity	GreInvia GrePatent	Add 1 to the number of green invention patents applied by enterprises, and then do the natural logarithm processing. Add 1 to the total number of green patents applied by enterprises, and then do the natural logarithm processing.
**Independent variable** Smart service degree	SmartSer	Ratio of the smart service keyword frequency of the enterprise to the sum of smart service keyword frequency of all enterprises in the same industry.
**Control variable** Enterprise size Cash holdings Asset-liability ratio Return on assets R&D intensity State-owned component Industry competitiveness Industrial policy	Size Cash Debt ROA RI State HHI IndPolicy	Natural log of the total number of employees. Ratio of monetary capital to total assets. Ratio of total liabilities to total assets. Ratio of net profit to total assets. Ratio of R&D expenses to revenue. The state-owned share of the total capital stock. Herfindahl-Hirschman Index: Sum of the squares of an enterprise’s share of revenue in an industry. Add 1 to the amount of policy subsidies received by enterprises, and then do the natural logarithm processing.

### 4.4 Model

According to the theoretical analysis, the baseline model in this study is constructed as follows:

$$Y_{i,t+1}=\alpha +\beta \times {SmartSer}_{i,t}+\delta \times {CV}_{i,t}+E_{i}$$ (3)

where *Y*_*i*,*t*+1_ is the dependent variable, including the green innovation quality and quantity of enterprise *i*, representing the sustainable green transformation of the enterprise; *SmartSer*_*i*,*t*_ is the independent variable and is the smart service degree of enterprise *i*; *CV*_*i*,*t*_ represents the eight selected control variables; and $E_{i}$ represents the residual.

## 5 Results and discussion

### 5.1 Descriptive statistics

Table 3 lists the descriptive statistics of the variables and shows the main characteristics of the sample enterprises. The mean and standard deviation of the smart service degree are 0.007 and 0.018, respectively, indicating obvious heterogeneity of smart service development among enterprises, which preliminarily indicates the necessity of studying the relationship between smart services and sustainable green transformation. As shown in Table 4, the correlation coefficient indicates that the correlation between independent variables and control variables is weak. Table 5 reports the evaluation results of the variance inflation factor (VIF). The maximum VIF of the independent variable and control variables is 2.372, which is far less than the critical value of 10. Therefore, the influence of multicollinearity on the model regression results is excluded.

**Table 3:** Descriptive statistics.

	Mean	S.D.	Min	Max	Median
**GreInvia**	1.003	1.208	0	6.562	0.693
**GrePatent**	1.226	1.296	0	6.730	1.099
**SmartSer**	0.007	0.018	0	0.201	0
**Size**	7.640	1.166	4.357	12.342	7.508
**Cash**	0.176	0.119	0.007	0.911	0.147
**Debt**	0.401	0.182	0.020	0.947	0.400
**ROA**	0.038	0.077	-0.458	0.383	0.040
**RI**	0.065	0.046	0	0.442	0.051
**State**	0.018	0.085	0	0.805	0
**HHI**	0.078	0.048	0.035	0.204	0.057
**IndPolicy**	17.640	1.544	0	22.968	17.606

**Table 4:** Correlation matrix.

	1	2	3	4	5	6	7	8	9	10	11
**1.GreInvia**	1										
**2.GrePatent**	0.962	1									
**3.SmartSer**	0.185	0.176	1								
**4.Size**	0.541	0.545	0.115	1							
**5.Cash**	0.017	0.001	-0.017	-0.103	1						
**6.Debt**	0.352	0.360	0.075	0.473	-0.281	1					
**7.ROA**	0.028	0.020	-0.025	0.025	0.267	-0.334	1				
**8.RI**	0.019	-0.003	0.086	-0.190	0.147	-0.229	-0.078	1			
**9.State**	0.104	0.092	0.079	0.065	0.059	0.071	0.046	0.022	1		
**10.HHI**	0.015	-0.005	0.168	0.176	-0.041	0.100	-0.051	-0.170	0.112	1	
**11.IndPolicy**	0.564	0.566	0.175	0.680	-0.042	0.346	0.035	0.103	0.093	0.052	1

**Table 5:** Variance inflation factor.

	VIF
**Independent variable** SmartSer	1.074
**Control variable**	
Size	2.372
Cash	1.150
Debt	1.670
ROA	1.289
RI	1.287
State	1.037
HHI	1.108
IndPolicy	2.152

### 5.2 Baseline regression

Table 6 reports the baseline regression results of smart services on sustainable green transformation. The degree of smart services has a significant positive effect (p<0.01) on the quality of green innovation (*Greinvia*) and the quantity of green innovation (*Grepatent*). The empirical results show that smart services promote both the quality and the quantity of green innovation, which verifies Hypothesis 1. In the context of the digital economy, big data, Industry 4.0 and other technologies play an important role in green innovation [61,62]. The results further show that smart service as a business model, enabled by new technologies, has a positive impact on the sustainable green transformation of enterprises.

**Table 6:** Results of baseline regression.

	GreInvia	GrePatent
	(1)	(2)
SmartSer	6.503*** (1.749)	6.683*** (1.874)
Size	0.286*** (0.040)	0.303*** (0.043)
Cash	0.826*** (0.273)	0.768*** (0.292)
Debt	1.085*** (0.215)	1.147*** (0.230)
ROA	0.695 (0.447)	0.595 (0.479)
RI	1.262* (0.749)	0.616 (0.802)
State	0.552 (0.361)	0.470 (0.387)
HHI	-1.838*** (0.669)	-2.610*** (0.717)
IndPolicy	0.235*** (0.029)	0.261*** (0.031)
Constant	-5.925*** (0.365)	-6.203*** (0.391)
F statistics	71.114***	71.846***
Adj.R^2^	0.394	0.397
Obs.	970	970

*p<0.10.

**p<0.05.

*** p<0.01.

### 5.3 Mediating mechanism

#### 5.3.1 Substitution effect of technology for capital

According to the principle and test procedure of the mediation effect, the mediation effect model is constructed in three steps as follows:

$$Y_{i,t+1}=\alpha_{1}+\beta_{1}\times {SmartSer}_{i,t}+\delta \times {CV}_{i,t}+E_{i}$$

$${INTER}_{i,t}=\alpha_{2}+\beta_{2}\times {SmartSer}_{i,t}+\delta \times {CV}_{i,t}+E_{i}$$

$$Y_{i,t+1}=\alpha_{3}+\beta_{3}\times {SmartSer}_{i,t}+\gamma \times {INTER}_{i,t}+\delta \times {CV}_{i,t}+E_{i}$$ (4)

where *INTER*_*i*,*t*_ is the mediator variable; other variables are defined in the same way as in Formula (3).

To test the substitution of technology for capital, we take the proportion of capital to the sum of technology and capital as a mediator variable. To ensure the equivalence of measurement, we use monetary value to measure each factor based on the financial disclosure data of listed enterprises. The summary of fixed assets, inventory and construction projects in progress is selected to measure the total capital. On this basis, the amount of fixed assets related to smart services (such as electronic equipment, automation equipment, communication equipment, management equipment, etc.) is deducted to finally form the capital factor measurement. Technology is measured by the book value of intangible assets such as software, technical systems, platforms and networks related to smart services and the expense of R&D. Therefore, the calculation formula of the substitution of technology for capital is:

$${KTS}_{i,t}=\frac{K_{i,t}-{SmartSerK}_{i,t}}{{(K}_{i,t}-{SmartSerK}_{i,t})+SmartSerT_{i,t}}$$ (5)

where *KTS*_*i*,*t*_ represents the substitution of technology for capital; *K*_*i*,*t*_ is capital; *SmartSerK*_*i*,*t*_ is fixed assets related to smart services; and *SmartSerT*_*i*,*t*_ is smart service technology.

Table 7 shows the results of the test for the substitution effect of technology for capital. The results in Column (1) show that the estimated coefficient of the smart service degree is significantly negative (p<0.01), indicating that with the development of smart services, the ratio of capital decreases significantly, and capital is significantly substituted by technology. The results in Columns (2)-(3) show that the estimated coefficients of the variable *KTS* pass the negative significance test (p<0.01). This indicates that, as the ratio of capital decreases, capital is substituted by technology, which has a positive role in promoting the sustainable green transformation of enterprises. Furthermore, bootstrap test results show that the 95% confidence interval of Bootstrap = 5000 for indirect effect does not contain zero value, and the mediation effect accounts for 8.6% and 10.2% of the total effect, respectively, indicating that production factor substation is an effective mediator variable. Confidence intervals are located to the right of zero, indicating that the mediation effect is positive. Information technology can be an effective substitute for capital [63], especially when the cost of technology is reduced with development [64]. The above results show that smart services positively affect the sustainable green transformation of enterprises through the substitution of technology for capital, which verifies Hypothesis 2.

**Table 7:** Test results of mediating mechanism (1).

	Substitution of technology for capital			Substitution of labor for capital		
	KTS	GreInvia	GrePatent	KLS	GreInvia	GrePatent
	(1)	(2)	(3)	(4)	(5)	(6)
SmartSer	-0.414*** (0.111)	5.942*** (1.756)	6.273*** (1.885)	-0.192*** (0.051)	5.861*** (1.752)	6.159*** (1.881)
KTS		-1.356*** (0.506)	-0.991* (0.543)			
KLS					-3.466*** (1.098)	-2.869** (1.178)
Size	-0.002 (0.003)	0.283*** (0.040)	0.301*** (0.043)	0.001 (0.001)	0.291*** (0.040)	0.308*** (0.043)
Cash	-0.138*** (0.017)	0.639** (0.281)	0.632** (0.301)	-0.046*** (0.008)	0.668** (0.276)	0.638** (0.296)
Debt	0.032** (0.014)	1.128*** (0.215)	1.178*** (0.230)	0.007 (0.006)	1.096*** (0.214)	1.154*** (0.230)
ROA	-0.073** (0.028)	0.596 (0.447)	0.522 (0.480)	-0.049*** (0.013)	0.520 (0.448)	0.448 (0.481)
RI	-0.725*** (0.048)	0.278 (0.832)	-0.102 (0.893)	-0.188*** (0.022)	0.604 (0.772)	0.071 (0.829)
State	0.016 (0.023)	0.574 (0.360)	0.486 (0.386)	0.006 (0.011)	0.518 (0364)	0.422 (0.390)
HHI	0.178*** (0.043)	-1.597** (0.673)	-2.434*** (0.722)	0.047** (0.020)	-1.656** (0.668	-2.453*** (0.717)
IndPolicy	-0.005** (0.002)	0.229*** (0.029)	0.257*** (0.031)	-0.003*** (0.001)	0.224*** (0.029)	0.252*** (0.031)
Constant	1.046*** (0.023)	-4.506*** (0.642)	-5.167*** (0.689)	1.037*** (0.011)	-2.322*** (1.195)	-3.222** (1.283)
Adj.R^2^	0.364	0.398	0.398	0.207	0.400	0.400
Bootstrap upper		0.118	0.003		0.177	0.089
Bootstrap lower		0.172	1.003		1.322	1.204
Obs.	970	970	970	970	970	970

*p<0.10.

**p<0.05.

*** p<0.01.

#### 5.3.2 Substitution effect of labor for capital

To test the substitution of labor for capital, the proportion of capital to the sum of the substitution labor factor and capital (*KTS)* is taken as a mediator variable. According to the theoretical analysis of this paper, labor indirectly substitutes capital through data in addition to direct substitution. Since smart data are formed after interacting with labor, we use the corresponding part of labor that synergizes with data as its proxy variable. The labor factor that substitutes the capital factor is usually the advanced labor factor. Based on the payroll payable, this paper makes a comprehensive calculation of labor by combining the proportion of employees with a bachelor’s degree or above, as well as data on the average salary of positions with different degrees from the Chinese Enterprises Recruitment Salary Report. Therefore, the calculation formula of the substitution of labor for capital is:

$${KLS}_{i,t}=\frac{K_{i,t}-{SmartSerK}_{i,t}}{\left(K_{i,t}-{SmartSerK}_{i,t})+({Edu}_{i,t}\times Pay)\times L_{i,t}/(1-{Edu}_{i,t}+{Edu}_{i,t}\times Pay\right)}$$ (6)

The test results in Columns (4)—(6) of Table 7 indicate that smart services improve the substitution of labor for capital, thus positively affecting the sustainable green transformation of enterprises. The mediation effect accounts for 6.5% and 8.2% of the total effect, respectively. The results show that smart services promote enterprise sustainable green transformation through the substitution of labor for capital. Hypothesis 3 passes the test. Data cannot directly affect the enterprise, but they can promote the formation of creativity and knowledge, which can guide the improvement of production and innovation [65,66]. This result shows that synergy with labor and data can reduce capital input, which can further affect the green transformation of enterprises.

#### 5.3.3 Human resource upgrading effect

The human capital degree (*HR_degree)* is used as the mediator variable to test the effect of human resource upgrading and is measured by the proportion of employees with a bachelor’s degree or above. The results in Table 8 show that smart services shift the demand for labor from low to high, consistent with the findings of Jason and Robert [67]. The upgrading of human resources is the mediator between smart services and sustainable green transformation. The bootstrap test shows that the mediation effect accounts for 38.8% and 36.6% of the total effect, respectively. The test results confirm Hypothesis 4.

**Table 8:** Test results of mediating mechanism (2).

	Human resources upgrading effect		
	HR_degree	GreInvia	GrePatent
	(1)	(2)	(3)
SmartSer	1.646*** (0.296)	3.864** (1.725)	4.080** (1.859)
HR_degree		1.556*** (0.186)	1.509*** (0.201)
Size	-0.059*** (0.007)	0.373*** (0.041)	0.388*** (0.044)
Cash	0.119** (0.047)	0.681** (0.267)	0.614** (0.288)
Debt	0.149*** (0.037)	0.906*** (0.212)	0.962*** (0.229)
ROA	0.203*** (0.076)	0.418 (0.440)	0.309 (0.474)
RI	1.443*** (0.127)	-1.052 (0.777)	-1.642* (0.837)
State	0.238*** (0.061)	0.164 (0.353)	0.092 (0.380)
HHI	-0.097 (0.114)	-1.700*** (0.654)	-2.474*** (0.705)
IndPolicy	0.043*** (0.005)	0.169*** (0.029)	0.198*** (0.031)
Constant	-0.222*** (0.062)	-5.595*** (0.357)	-5.884*** (0.385)
Adj.R^2^	0.320	0.436	0.431
Bootstrap upper		3.572	3.458
Bootstrap lower		1.306	1.238
Obs.	970	970	970

*p<0.10.

**p<0.05.

*** p<0.01.

### 5.4 Heterogeneity analysis

Enterprises’ external macro environment and their own micro characteristics are distinct, which may affect the sustainable green transformation effect of smart services. We explore the possible heterogeneity based on the regional new infrastructure, environmental hazard degree and property rights of enterprises.

#### 5.4.1 Regional new infrastructure

Based on the information of the enterprise registration place and the progress of the national smart city pilot program, we divide the samples into new infrastructure areas and nonnew infrastructure areas for group regression. The results in Table 9 show that the estimated coefficients of the smart service degree are significantly positive (p<0.01) in the new infrastructure area; the estimated coefficient of the smart service degree is positive in the regions without new infrastructure investment, but it does not pass the significance test (p>0.10).

**Table 9:** Results of regional new infrastructure heterogeneity.

	New infrastructure areas		Nonnew infrastructure areas	
	GreInvia	GrePatent	GreInvia	GrePatent
	(1)	(2)	(3)	(4)
SmartSer	9.073*** (2.298)	9.009*** (2.462)	1.886 (2.547)	2.881 (2.745)
CVs	Yes	Yes	Yes	Yes
Adj.R^2^	0.403	0.402	0.361	0.373
Obs.	732	732	238	238

*p<0.10.

**p<0.05.

*** p<0.01.

The possible reasons for this heterogeneous result include the following two aspects. From the dimension of data volume [68], the new infrastructure promotes the rapid growth of the data scale, enabling enterprises to obtain the data scale effect. From the dimension of data accessibility [68], the new infrastructure improves the level of data openness and connectivity, enabling enterprises to obtain network effects. Therefore, the value of the data has been greatly improved, enabling smart data to fully release the environmental dividend of the production factor substitution effect in the region. In contrast, in regions where investment in new infrastructure is lagging, the scale and network effects of data are limited, and thus, the sustainable green transformation effect of smart services may not play out. This result shows that the layout of new infrastructure construction should be rationally arranged, coordinated and balanced, and new infrastructure in uncovered areas should be added as soon as possible. Then, the spillover of new infrastructure can be fully played through the scale effect and network effect of smart data to promote the manufacturing industry to achieve energy conservation and emission reduction through smart services.

#### 5.4.2 Environmental hazards

According to the list of key polluters published by the environmental protection department, the environmental hazard degree of enterprises is classified. We divide the samples into key polluters and nonkey polluters for grouping regression. As shown in Table 10, the grouping regression results show that the development of smart services in key polluters has a higher effect on green innovation, indicating that enterprises with serious environmental hazards have a stronger promoting effect on sustainable green transformation through smart services compared with nonkey polluters.

**Table 10:** Results of environmental hazard heterogeneity.

	Key polluters		Nonkey polluters	
	GreInvia	GrePatent	GreInvia	GrePatent
	(1)	(2)	(3)	(4)
SmartSer	8.738*** (3.263)	7.027** (3.403)	4.362** (2.078)	5.371** (2.252)
CVs	Yes	Yes	Yes	Yes
Adj.R^2^	0.494	0.510	0.301	0.296
Obs.	289	289	681	681

*p<0.10.

**p<0.05.

*** p<0.0.

A possible reason for this result is that enterprises with more serious environmental problems are subject to a greater intensity of pressure from various parties. Due to the higher cost of regulation and survival crises [69,70], more attention is given to the innovation and development of business models to break through the original development model and achieve sustainable green transformation. In addition, this heterogeneous result indicates that the promoting effect of smart services on sustainable green transformation meets the requirements of environmental protection and emission reduction.

#### 5.4.3 Property right attribute

According to the stock rights of enterprises, the samples are divided into state-owned enterprises and nonstate-owned enterprises for grouping regression. As shown in Table 11, the grouped regression results show that the coefficients of the smart services degree of state-owned enterprises are larger than those of nonstate-owned enterprises, indicating that smart services have a stronger promoting effect on the sustainable green transformation of state-owned enterprises.

**Table 11:** Results of property right attribute heterogeneity.

	state-owned		nonstate-owned	
	GreInvia	GrePatent	GreInvia	GrePatent
	(1)	(2)	(3)	(4)
SmartSer	7.922** (3.594)	8.701** (3.940)	4.995** (2.023)	5.025** (2.238)
CVs	Yes	Yes	Yes	Yes
Adj.R^2^	0.464	0.485	0.322	0.325
Obs.	192	192	778	778

*p<0.10.

**p<0.05.

*** p<0.01.

Although private enterprises have a strong sense of innovation, quick decision-making and more flexible operation, they often lack strategic research and long-term consideration, and they give more attention to the speed of development and ignore the accumulation of resources and capabilities. In contrast, state-owned enterprises have a greater sense of social responsibility due to their political attributes and often have clear green development strategic planning. Broader government objectives and long-term policy choices have a positive impact on firm innovation [71]. It is easier to obtain government project support and resources for state-owned enterprises, which can promote their corporate performance [72]. The management and operation of state-owned enterprises are standardized. Therefore, they can better realize smart service development. By realizing the substitution of the production factor and upgrading human resources, the sustainable green transformation effect can be fully brought into play in state-owned enterprises.

### 5.5 Robustness checks

#### 5.5.1 Variable substitution check

First, we replace the independent variable with smart service decision (*SmartSer_dum*), which is the dummy variable of whether an enterprise develops a smart service business. If the enterprise’s business includes smart services, the value is 1; otherwise, the value is 0. The results in Columns (1) and (2) of Table 12 show that the effect of the *SmartSer* variable on the two dependent variables is still significantly positive (p<0.01).

**Table 12:** Robustness test.

	Independent variable substitution		Dependent variable substitution		IV- 2SLS	
	GreInvia	GrePatent	GreInvia	GrePatent	GreInvia_g	GrePatent_g
	(1)	(2)	(3)	(4)	(5)	(6)
SmartSer			5.427*** (1.554)	7.774*** (1.987)	25.111*** (8.470)	26.893*** (9.086)
SmartSer_dum	0.451*** (0.065)	0.498*** (0.069)				
Size	0.299*** (0.039)	0.318*** (0.042)	0.257*** (0.035)	0.339*** (0.045)	0.291*** (0.042)	0.309*** (0.045)
Cash	0.644** (0.269)	0.569** (0.287)	0.611** (0.242)	0.417 (0.310)	0.868*** (0.289)	0.814*** (0.310)
Debt	1.007*** (0.211)	1.059*** (0.226)	0.514*** (0.191)	1.334*** (0.244)	1.031*** (0.228)	1.088*** (0.245)
ROA	0.808* (0.440)	0.719 (0.470)	-0.242 (0.397)	0.246 (0.508)	0.699 (0.473)	0.599 (0.507)
RI	1.115 (0.735)	0.435 (0.785)	1.955*** (0.666)	1.478* (0.851)	0.507 (0.860)	-0.203 (0.922)
State	0.647* (0.354)	0.570 (0.379)	0.443 (0.321)	0.536 (0.410)	0.384 (0.389)	0.288 (0.417)
HHI	-0.556 (0.659)	-1.228* (0.705)	-0.881 (0.595)	-2.240*** (0.760)	-3.047*** (0.888)	-3.924*** (0.953)
IndPolicy	0.200** (0.029)	0.222*** (0.031)	0.189*** (0.026)	0.261*** (0.033)	0.202*** (0.034)	0.225*** (0.036)
Constant	-5.642*** (0.361)	-5.877** (0.386)	-5.130*** (0.324)	-6.265*** (0.415)	-5.354*** (0.462)	-5.583*** (0.495)
F statistics	77.522***	79.069***	57.884***	74.178***	63.217***	63.791**
Adj.R^2^	0.415	0.420	0.346	0.405	0.323	0.324
DW (p value)					0.017	0.016
Cragg-Donald F statistics					48.057 [8.96]	48.057 [8.96]
Obs.	970	970	970	970	970	970

Note: The thresholds for Stock-Yogo at the 5% significance level are shown in square brackets.

*p<0.10.

**p<0.05.

*** p<0.01.

Then, we replace the dependent variables. The number of green invention patents granted (*GreInvia_g*) and the total number of green patents granted (*GrePatent_g*) are taken as the proxy variables of sustainable green transformation, and the corresponding index of application volume is replaced. The test results are shown in rows (3) and (4) of Table 12. The smart service degree (*SmartSer*) has a significant positive effect on the number of green invention patents granted (GreInvia_g) and the total number of green patents granted (GrePatent_g) and passes the significance test (p<0.01).

In summary, the regression results after the substitution of independent and dependent variables show that the results of the baseline regression remain robust.

#### 5.5.2 Endogeneity check

To address the endogeneity problem, the IV and 2SLS methods are used to test endogeneity. According to the principle of instrumental variables, the selection of instrumental variables should meet two conditions: correlation and externality. Based on the BLP principle [73], this paper constructs instrumental variables from the enterprise size data of all other enterprises in the same industry except enterprise *i*, which are related to the smart service of enterprise *i* but have no direct correlation with the sustainable green transformation performance of enterprise *i*. On the one hand, the smart service degree of enterprise *i* is affected by competitive substitutes. Enterprise *i* may respond to the smart service measures of competitors through changes such as smart service strategy to gain advantages or avoid falling behind competitors [11]. On the other hand, the smart service degree, size, asset-liability ratio of enterprise *i*, and other regression factors in the model may affect the sustainable green transformation of enterprise *i*. However, the process of sustainable green transformation of enterprise *i* will not be directly affected by the size of enterprise *j*. Therefore, the instrumental variable for this study is to calculate the sum of enterprise size (represented by total assets) of all enterprises developing smart services in the same industry except itself. The formula is:

$${IV}_{i,t}=(\sum_{k=1}^{n-1}{{Assest}_{k,t}\times Smaser\_dum}_{k,t})/(n-1)$$ (7)

where *Assest*_*k*,*t*_ represents the total assets of enterprise *k*; *Smaser*_*dum*_*k*,*t*_ represents the smart service decision of enterprise *k*; and *n* represents the total number of enterprises in the industry to which enterprise *i* belongs.

The results of Columns (5) and (6) in Table 12 show that the p values of the Durbin-Wu-Hausman test are 0.017 and 0.016, respectively. This shows that the hypothesis of exogenous independent variables is rejected at the 5% significance level, demonstrating the applicability of the instrumental variable method. The value of the Cragg-Donald F statistic is 48.057, which is greater than the critical value of 8.96 of Stock-Yogo at the 5% significance level. This shows that the hypothesis of weak identification of the instrumental variable is rejected and that the instrumental variable is valid. On the premise of effectively controlling the possible endogeneity problems, the estimated coefficients of green innovation quality and quantity both pass the positive significance test (p<0.01), which proves that the above results of the baseline regression are robust.

## 6. Conclusions and implications

Smart services have a significant positive impact on the quality and quantity of green innovation and they promote the sustainable green transformation of enterprises, especially for enterprises with high environmental hazards that are eager to break out of their traditional development model. As a kind of service model innovation, its development follows market rules and has business model incentives. Smart services are an effective endogenous driving force of sustainable green transformation for enterprises.

The development of smart services leads to the effective substitution of factors of production, as well as the upgrading of human resources, through which smart services promote the sustainable green transformation of enterprises. Among them, human resources upgrading has the largest mediating effect.

The heterogeneity effect study shows that investment in new infrastructure construction contributes to the successful sustainable green transformation of smart service enterprises. In regions not covered by new infrastructure, smart services fail to have a significant impact on sustainable green transformation. Despite the large number of private enterprises in China, limited by their strategies and capabilities, the sustainable green transformation effect of smart services is not as good as that of state-owned enterprises.

The study has important policy and managerial implications. Taking Chinese listed enterprises as an example, the promoting effect of smart services on sustainable green transformation is verified in the study. Enterprises can achieve sustainable green transformation by actively changing their development mode. This solves the problem of external policy failure from the source of the driving force and resolves the dilemma between development and emission reduction. Therefore, the government can take the development of advanced manufacturing as one of the important strategic guidelines for green development. The positive support role of national and industrial funds for the development of smart services should be increased.

From the perspective of the external environment, the government needs to rationally arrange the construction of new infrastructure so that the spillover can be fully released through the scale effect and network effect. The market-based allocation mechanism of production factors needs to be further improved to reduce the market-based acquisition costs of technology and labor. Especially given the new data factor, it is necessary to establish and improve the standards and norms of the collection, storage and transaction of data at the institutional level. Then, the development of data can be sustainable and can fully release the substitution effect of factors of production. In addition, to better promote sustainable green transformation, attention should be given to the cultivation and introduction of talent needed for the high-quality development of the manufacturing industry.

For enterprises and managers, the important role of smart services in the coordinated development of the economy and environment should be fully recognized and utilized. Enterprises should actively build or integrate into the ecological industry chain of smart services. Enterprises can maximize the substitution effect of production factors by means of technology and knowledge spillover within the ecological chain and through the open sharing of data in a wider range. By being more proactive in achieving sustainable green transformation through developing smart services, enterprises can balance their economic and environmental performance to improve their comprehensive competitiveness.

The study takes listed manufacturing enterprises as samples, and the applicability and extensibility of the research conclusions to nonlisted enterprises are still unclear. Further research can be expanded to contribute to the evidence on the relationship between smart services and sustainable green transformation based on other new measurement methods of smart services. In addition, since the sample enterprises are from China, the conclusion is mainly aimed at China and other emerging economies. Therefore, future studies can further compare the green transformation effect of smart services in emerging economies and developed countries, which can carry important research value.

## Abbreviations

OECD: Organization for Economic Co-operation and Development
GTFP: green total factor productivity
AHP: analytic hierarchy process
VIF: variance inflation factor
IV: instrumental variable
2SLS: two-stage least square
AI: artificial intelligence

## References

[1] Agrawal VV, Bellos I. The Potential of Servicizing as a Green Business Model. Manage Sci. 2017;63(5):1545–62. 10.1287/mnsc.2015.2399.

[2] Missimer M, Mesquita PL. Social Sustainability in Business Organizations: A Research Agenda. Sustainability-Basel. 2022;14(5):2608. 10.3390/su14052608.

[3] Kevin DB, Roger B, Judy Z. Environmental sustainability: a value cycle research agenda. Prod Plan Control. 2012;23(2–3):105–19. 10.1080/09537287.2011.591621.

[4] Xin L, Ahmad M, Murshed M. Toward next-generation green solar cells and environmental sustainability: impact of innovation in photovoltaic energy generation, distribution, or transmission-related technologies on environmental sustainability in the United States. Environmental science and pollution research international. 2022;29(59):89662–80. doi: 10.1007/s11356-022-21953-w 35857166

[5] Ahmed Z, Ahmad M, Murshed M, Ibrahim SM, Mahmood H, Abbas S. How do green energy technology investments, technological innovation, and trade globalization enhance green energy supply and stimulate environmental sustainability in the G7 countries? Gondwana Res. 2022;112:105–15. 10.1016/j.gr.2022.09.014.

[6] Yuan X, Murshed M, Khan S. Does the depth of the Financial Markets matter for establishing Green Growth? Assessing Financial sector’s potency in decoupling Economic Growth and Environmental Pollution. Evaluation Rev. 2022;0(0). doi: 10.1177/0193841X221145777 36530001

[7] Jahanger A, Yu Y, Hossain MR, Murshed M, Balsalobre-Lorente D, Khan U. Going away or going green in NAFTA nations? Linking natural resources, energy utilization, and environmental sustainability through the lens of the EKC hypothesis. Resour Policy. 2022;79:103091. 10.1016/j.resourpol.2022.103091.

[8] Abro AA, Alam N, Murshed M, Mahmood H, Musah M, Rahman AKMA. Drivers of green growth in the Kingdom of Saudi Arabia: can financial development promote environmentally sustainable economic growth? Environmental science and pollution research international. 2022:1–17. 10.1007/s11356-022-23867-z. 36327073

[9] Chiehyeon L, Paul PM. Data-Driven Understanding of Smart Service Systems Through Text Mining. Serv Sci. 2018;10(2):154–80. 10.1287/serv.2018.0208.

[10] Langley DJ. Digital Product-Service Systems: The Role of Data in the Transition to Servitization Business Models. Sustainability-Basel. 2022;14(3):1303. 10.3390/su14031303.

[11] Glen A, Ralph L. Four strategies for the age of smart services. Harvard Bus Rev. 2005;83(10):131–45. 16250631

[12] Marcus F, David H, Adrian H, Christian J, Christoph K, Axel W. A taxonomy and archetypes of smart services for smart living. Electron Mark. 2020;30:131–49. 10.1007/s12525-019-00384-5.

[13] Oberlaender AM, Roeglinger M, Rosemann M, Kees A. Conceptualizing business-to-thing interactions—A sociomaterial perspective on the Internet of Things. Eur J Inform Syst. 2018;27(4):486–502. 10.1080/0960085X.2017.1387714.

[14] Graetz G, Michaels G. Robots at Work. Rev Econ Stat. 2018;100(5):753–68. 10.1162/rest_a_00754.

[15] Daron A, Pascual R. The Race between Man and Machine: Implications of Technology for Growth, Factor Shares, and Employment. Am Econ Rev. 2018;108(6). 10.3386/w22252.

[16] Porter ME, Heppelmann JE. How Smart, Connected Products Are Transforming Companies. Harvard Bus Rev. 2015;93(10):96–114.

[17] Alcayaga A, Wiener M, Hansen EG. Towards a framework of smart-circular systems: An integrative literature review. J Clean Prod. 2019;221:622–34. 10.1016/j.jclepro.2019.02.085.

[18] Haarstad H, Wathne MW. Are smart city projects catalyzing urban energy sustainability? Energ Policy. 2019;129:918–25. 10.1016/j.enpol.2019.03.001.

[19] Guo Q, Wang Y, Dong X. Effects of smart city construction on energy saving and CO2 emission reduction: Evidence from China. Appl Energ. 2022;313:118879. 10.1016/j.apenergy.2022.118879.

[20] Tan Y, Md K. Does smart city policy lead to sustainability of cities? Land Use Policy. 2018;73. 10.1016/j.landusepol.2018.01.034.

[21] Acemoglu D, Restrepo P. Automation and New Tasks: How Technology Displaces and Reinstates Labor. J Econ Perspect. 2019;33(2):3–29. 10.1257/jep.33.2.3.

[22] Popp DC. The effect of new technology on energy consumption. Resour Energy Econ. 2000;23(3):215–39. 10.1016/s0928-7655(00)00045-2.

[23] Boqiang L, Weisheng L. Estimation of energy substitution effect in China’s machinery industry—based on the corrected formula for elasticity of substitution. Energy. 2017;129:246–54. 10.1016/j.energy.2017.04.103.

[24] Xiaoling O, Wuxu Z, Gang D, Kerui D. Output elasticities and inter-factor substitution: Empirical evidence from the transportation sector of Shanghai. J Clean Prod. 2018;202:969–79. 10.1016/j.jclepro.2018.08.188.

[25] Rainer A, Haluk D, Jan FE, Anne M, Alfred W. Smart services: The move to customer orientation. Electron Mark. 2019;29(1):1–6. 10.1007/s12525-019-00338-x.

[26] Kampker A, Stich V, Jussen P, Moser B, Kuntz J. Business Models for Industrial Smart Services—The Example of a Digital Twin for a Product-Service-System for Potato Harvesting. 11th Cipp Conference on Industrial Product-Service Systems. 2019;83:534–40. 10.1016/j.procir.2019.04.114.

[27] Kaňovská L, Tomášková E. Drivers for Smart Servitization in Manufacturing Companies. Agris on-line Papers in Economics and Informatics. 2018;10(3):57–68. 10.7160/aol.2018.100305.

[28] Rapaccini M, Adrodegari F. Conceptualizing customer value in data-driven services and smart PSS. Comput Ind. 2022;137. 10.1016/j.compind.2022.103607.

[29] Daniel B, Martin M, Christian J. Information systems for smart services. Inf Syst E-Bus Manag. 2017;15(4):103607. 10.1007/s10257-017-0365-8.

[30] Daniel B, Oliver M, Martin M, Jan M, Jan VB. Conceptualizing smart service systems. Springer Berlin Heidelberg. 2019;29(1):7–18. 10.1007/s12525-017-0270-5.

[31] Alexandra M. Editorial Column—Smart Things as Service Providers: A Call for Convergence of Disciplines to Build a Research Agenda for the Service Systems of the Future. Serv Sci. 2015;7(1):ii–v. 10.1287/serv.2014.0090.

[32] Robin K, Andreas J, Matthias S, Jan ML. Value Co-Creation in Smart Services: A Functional Affordances Perspective on Smart Personal Assistants. J Assoc Inf Syst. 2021;22(2):418–58. 10.17705/1jais.00667.

[33] Porter ME, Heppelmann JE. How Smart, Connected Products Are Transforming Competition. Harvard Bus Rev. 2014;92(11):64–88.

[34] Mikusz M. Towards an Understanding of Cyber-physical Systems as Industrial Software-Product-Service Systems. Procedia CIRP. 2014;16:385–9. http://doi.org/10.1016/j.procir.2014.02.025.

[35] Freitag M, Haemmerle O. Agile Guideline for Development of Smart Services in Manufacturing Enterprises with Support of Artificial Intelligence. ADVANCES IN PRODUCTION MANAGEMENT SYSTEMS: THE PATH TO DIGITAL TRANSFORMATION AND INNOVATION OF PRODUCTION MANAGEMENT SYSTEMS. 2020;591:645–52. 10.1007/978-3-030-57993-7_73.

[36] Shubhangini R, Surya PS. Connecting circular economy and industry 4.0. Int J Inform Manage. 2019;49:98–113. 10.1016/j.ijinfomgt.2019.03.002.

[37] Kannan G, Ahmad J, Vahid N. Designing a sustainable supply chain network integrated with vehicle routing: A comparison of hybrid swarm intelligence metaheuristics. Computers and Operations Research. 2019;110:220–35. 10.1016/j.cor.2018.11.013.

[38] Alberto V. Smartmentality: The Smart City as Disciplinary Strategy. Urban Stud. 2014;51(5):883–98. 10.1177/0042098013494427.

[39] Haarstad H. Constructing the sustainable city: examining the role of sustainability in the "smart city’ discourse. J Environ Pol Plan. 2017;19(4):423–37. 10.1080/1523908X.2016.1245610.

[40] Ahvenniemi H, Huovila A, Pinto-Seppa I, Airaksinen M. What are the differences between sustainable and smart cities? Cities. 2017;60(A):234–45. 10.1016/j.cities.2016.09.009.

[41] Tan Y, Sang HL. Korean ubiquitous-eco-city: A smart-sustainable urban form or a branding hoax? Technological Forecasting & Social Change. 2014;89:100–14. 10.1016/j.techfore.2013.08.034.

[42] Sofia TS. A Model Korean Ubiquitous Eco-City? The Politics of Making Songdo. J Urban Technol. 2013;20(1):39–55. 10.1080/10630732.2012.735409.

[43] Jason F, Robert S. AI and the Economy. Innovation Policy and the Economy. 2019;19:161–91. 10.1086/699936.

[44] Bessen J. Automation and jobs: when technology boosts employment. Econ Policy. 2019;34(100):589–626. 10.2139/ssrn.2935003.

[45] Berndt ER, Wood DO. Technology, Prices, and the Derived Demand for Energy. The Review of Economics and Statistics. 1975;57(3):259–68. 10.2307/1923910.

[46] Andy N. Exploring the financial consequences of the servitization of manufacturing. Oper Manage Res. 2009;1(2):103–18. 10.1007/s12063-009-0015-5.

[47] Arnold T. Eight types of product–service system: eight ways to sustainability? Experiences from SusProNet. Bus Strateg Environ. 2004;13(4):246–60. 10.1002/bse.414.

[48] Arend MG, Franke T. The Role of Interaction Patterns with Hybrid Electric Vehicle Eco-Features for Drivers’ Eco-Driving Performance. Hum Factors. 2017;59(2):314–27. doi: 10.1177/0018720816670819 27702984

[49] Selcuk S. Predictive maintenance, its implementation and latest trends. P I Mech Eng B-J Eng. 2017;231(9):1670–9. 10.1177/0954405415601640.

[50] Spring M, Araujo L. Product biographies in servitization and the circular economy. Ind Market Manag. 2017;60:126–37. 10.1016/j.indmarman.2016.07.001.

[51] Wiegard R, Breitner MH. Smart services in healthcare: A risk-benefit-analysis of pay-as-you-live services from customer perspective in Germany. Electron Mark. 2019;29(1):107–23. 10.1007/s12525-017-0274-1.

[52] Marquardt K. Smart services—characteristics, challenges, opportunities and business models. Proceedings of The International Conference on Business Excellence. 2017;11(1):789–801. 10.1515/picbe-2017-0084.

[53] Georg G, Guy M. Robots at Work. Rev Econ Stat. 2018;100(5):753–68. 10.1162/rest_a_00754.

[54] James B. Automation and jobs: when technology boosts employment. Econ Policy. 2019;34(100):589–626. 10.2139/ssrn.2935003.

[55] Manjul G, Joey FG. Toward the development of a big data analytics capability. Inform Manage-Amster. 2016;53(8):1049–64. 10.1016/j.im.2016.07.004.

[56] Erik B, Tom M, Daniel R. What Can Machines Learn and What Does It Mean for Occupations and the Economy? AEA Papers and Proceedings. 2018;108:43–7. 10.1257/pandp.20181019.

[57] Acemoglu D, Restrepo P. Automation and New Tasks: How Technology Displaces and Reinstates Labor. J Econ Perspect. 2019;33(2):3–30. 10.1257/jep.33.2.3.

[58] Gil D, Ferrandez A, Mora-Mora H, Peral J. Internet of Things: A Review of Surveys Based on Context Aware Intelligent Services. Sensors-Basel. 2016;16(7):1069. doi: 10.3390/s16071069 27409623 PMC4970116

[59] Bello O, Zeadally S. Toward efficient smartification of the Internet of Things (IoT) services. Future Gener Comp Sy. 2019;92:663–73. 10.1016/j.future.2017.09.083.

[60] Kim TH, Ramos C, Mohammed S. Smart City and IoT. Future Gener Comp Sy. 2017;76:159–62. 10.1016/j.future.2017.03.034.

[61] El-Kassar A, Singh SK. Green innovation and organizational performance: The influence of big data and the moderating role of management commitment and HR practices. Technol Forecast Soc. 2019;144:483–98. 10.1016/j.techfore.2017.12.016.

[62] Muhammad FM, Suman T, Monika P, Mobashar M, Raja ZRMR. How Industry 4.0 technologies and open innovation can improve green innovation performance? Management of Environmental Quality: An International Journal. 2021;32(5):1007–22. 10.1108/MEQ-11-2020-0266.

[63] Sanjeev D, Chung-ki M. The Substitution of Information Technology for Other Factors of Production: A Firm Level Analysis. Manage Sci. 1997;43(12):1660–75. 10.2307/2634534.

[64] Chun H, Mun S. Substitutability and Accumulation of Information Technology Capital in U.S. Industries. South Econ J. 2006;72(4):1002–15. 10.1002/J.2325-8012.2006.TB00750.X.

[65] Rong K. Research agenda for the digital economy. Journal of Digital Economy. 2022;1(1). 10.1016/j.jdec.2022.08.004.

[66] Abdul-Nasser E, Sanjay KS. Green innovation and organizational performance: The influence of big data and the moderating role of management commitment and HR practices. Technological Forecasting & Social Change. 2019;144:483–98. 10.1016/j.techfore.2017.12.016.

[67] Jason F, Robert S. AI and the Economy. Innovation Policy and the Economy. 2019;19(1):161–91. 10.1086/699936.

[68] Chakravorti B, Bhalla A, Chaturvedi RS. Which Countries Are Leading the Data Economy? Harvard Bus Rev. 2019;1:2–8.

[69] Nicolò B. Investigating the impacts of technological position and European environmental regulation on green automotive patent activity. Ecol Econ. 2015;117. 10.1016/j.ecolecon.2015.06.017.

[70] Jaegul L, Francisco MV, David AH. Linking induced technological change, and environmental regulation: Evidence from patenting in the U.S. auto industry. Res Policy. 2011;40(9):1240–52. 10.1016/j.respol.2011.06.006.

[71] Chang S, Chung C, I, Mahmood SP. When and How Does Business Group Affiliation Promote Firm Innovation? A Tale of Two Emerging Economies. Organization Science. 2006;17(5):637–56. 10.2307/25146064.

[72] Daphne Y, Garry DB, Yuan L. Understanding Business Group Performance in an Emerging Economy: Acquiring Resources and Capabilities in Order to Prosper. J Manage Stud. 2005;42(1):183–206. 10.1111/j.1467-6486.2005.00493.x.

[73] Berry S, Levinsohn J, Pakes A. Automobile Prices in Market Equilibrium. Econometrica. 1995;63(4):841. 10.2307/2171802.

